# Gasdermin D (GSDM D) as a Potential Diagnostic Biomarker in Bladder Cancer: New Perspectives in Detection

**DOI:** 10.3390/cancers16244213

**Published:** 2024-12-18

**Authors:** Monika Gudowska-Sawczuk, Sara Pączek, Michał Olkowicz, Jacek Kudelski, Barbara Mroczko

**Affiliations:** 1 Department of Biochemical Diagnostics, Medical University of Bialystok, Waszyngtona 15A St., 15-269 Bialystok, Poland; barbara.mroczko@umb.edu.pl; 2 Department of Biochemical Diagnostics, University Hospital of Bialystok, Waszyngtona 15A St., 15-269 Bialystok, Poland; sara.paczek@uskwb.pl; 3 Department of Urology, Medical University of Bialystok, M. Skłodowskiej-Curie 24A St., 15-276 Białystok, Poland; m.olkowicz@yahoo.com (M.O.); jkudelski@op.pl (J.K.); 4 Department of Neurodegeneration Diagnostics, Medical University of Bialystok, Waszyngtona 15A St., 15-269 Bialystok, Poland

**Keywords:** gasdermin, GSDM D, bladder, cancer, tumour marker, diagnosis

## Abstract

Background: Traditional methods of bladder cancer (BC) diagnosis include clinical examination, imaging, urine tests, cystoscopy, and biopsy. Due to the complexity of detection, diagnostic markers of bladder cancer measured in blood are still being sought. The pathogenesis of BC is complex and depends on many factors. However, the available literature data suggest that gasdermin D (GSDM D) has an influence in the pathogenesis of cancers. This study is the first that assesses the significance and diagnostic utility of serum GSDM D in bladder cancer. Methods: A total of 80 serum samples were obtained and analysed from healthy volunteers (C) and bladder cancer patients. The cancer patients were further classified into two groups, low-grade (LG) and high-grade (HG) bladder cancer, according to the TNM classification. The serum levels of GSDM D, CEA, and CA19-9 were assessed following the manufacturer’s instructions using immunoassay techniques. Results: The concentrations of GSDM D were nearly twice as high in the serum of BC patients compared to controls. Additionally, the median of GSDM D concentration was notably elevated in LG and HG bladder cancer than in C. In the total study group, the GSDM D concentration correlated with CRP and CEA levels. The diagnostic sensitivity of GSDM D was higher than that of CEA, but slightly lower in comparison to CA19-9. Moreover, the negative predictive value of GSDM D was the highest among all tested markers. The highest positive predictive value and diagnostic accuracy were observed for CEA, while the accuracy of GSDM D was comparable. GSDM D had an AUC value of 0.741, which was similar to the AUC value of CEA. Conclusions: GSDM D in serum appears to be a valuable diagnostic biomarker, especially when its measurement is used in conjunction with other markers such as CEA. Its high sensitivity makes it effective for the early detection of bladder cancer.

## Simple Summary

Bladder cancer is a significant healthcare problem due to its increasing incidence and high morbidity and mortality rates. It affects a large number of people globally, and its management poses both clinical and economic challenges. Some studies have suggested that the expression of gasdermin D in bladder cancer cells could be associated with the progression of the disease. Elevated levels of gasdermin D expression has been observed to potentially correlate with more aggressive tumour features or poor prognosis. The role of gasdermin D in bladder cancer is still being explored, but it represents a potentially important player in both the immune response and cancer progression. Therefore, the aim of this study was to assess the significance and diagnostic utility of serum GSDM D in bladder cancer.

## 1. Introduction

Bladder cancer (BC) is a type of malignant tumour that develops in the cells that line the urinary bladder. It is one of the most common types of urinary tract cancer and stands as serious global health problem [1]. The most common histological type of BC is urothelial carcinoma, also known as transitional cell carcinoma (TCC). It accounts for over 90% of BC cases [2]. This cancer arises from the urothelial epithelium, which lines not only the bladder, but also areas such as the renal pelvis, ureters, and urethra. For this reason, the entire urinary system is examined during the diagnosis and treatment of BC. Other types of BC include squamous cell carcinoma (SCC) and adenocarcinoma [2,3]. The pathogenesis of BC is complex and depends on many genetic, environmental, and immunological factors. It is believed that one of the main factors that increases the risk of developing BC is smoking. The risk of tumour can also be increased by certain chemicals associated with various occupational exposures [4,5,6]. BC symptoms may vary depending on the stage and advancement of the disease [1,7]. However, unfortunately, early and non-specific signs of the disease are very often underestimated. Symptoms of BC may include haematuria, pain and burning when urinating, frequent urination, and a discomfort or pressure in the bladder. Pain in the urinary bladder area, or weight loss, weakness, and fatigue often appear only in the advanced stages of the disease. The effectiveness of treatment depends on the stage of advancement and individual patient factors. Hence, early detection and treatment can significantly improve the prognosis for BC patients [1,8]. Diagnosing BC, as with all cancers, is a complex process. It begins with an interview and a physical examination. Then it includes imaging methods such as abdominal ultrasound (USG), computed tomography (CT), and urography. However, one of the most important tests in diagnosing BC is cystoscopy, which involves visual assessment of the mucosa lining of the urinary bladder using a cystoscope inserted through the urethra [9]. The diagnostic process is also supported by laboratory tests, including cytological evaluation of urine sediment and circulating tumour markers such as bladder tumour antigen (BTA) and nuclear matrix protein 22 (NMP22) [10,11,12]. There are also scientific studies in which serum carbohydrate antigen 19-9 (CA19-9) is elevated in all patients with advanced BC, despite it being a first-line marker for pancreatic cancer [13]. Moreover, in bladder cancer, overexpression of carcinoembryonic antigen (CEA) has also been noted; however, CEA is primarily expressed in the colorectal epithelium and the surface epithelium of the gastric mucosa [14].

The development of bladder cancer is complex and influenced by a variety of factors [7,15]. Some evidence suggests that gasdermins (GSDMs) play a role in the pathogenesis of many tumours, including BC [16]. The GSDM family consists of six proteins, including GSDM A, -B, -C, -D, -E, and Pejvakin (PJVK) [17]. Each of them is composed of two parts; one initiates pyroptosis, and the other is responsible for binding the first one to prevent it from killing the cell. Activation of GSDM occurs by separating these two parts with the help of proteases. GSDM exerts its action by inducing a phenomenon called pyroptosis [17,18,19]. This is a process different than apoptosis and occurs as a result of infection of the cell with viruses or bacteria. As a result, the cell membrane ruptures and the cell contents escape, which initiates an inflammatory reaction [20]. Recent research suggests that GSDMs influence the immune response against cancer. Their role in regulating the inflammatory response may influence the activation of immune cells to fight cancer cells [21]. One of the best-known proteins of the GSDM family is gasdermin D (GSDM D). Its expression has been observed in, for example, immune cells and the epithelial cells of the stomach, oesophagus, and intestines. GSDM D plays an important role in in the human body’s immune response, in which it is an essential protein that activates cells of the innate immune response, i.e., monocytes and macrophages, among others, during microbial infection, causing their osmotic lysis as a result of pyroptosis [16,22]. Although it is mainly associated with resistance to infection, some studies suggest that the induction of pyroptosis in cancer cells may lead to their death. It is a complex process characterised by a strong inflammatory response and the release of pro-inflammatory cytokines and other inflammatory mediators. The best known, GSDM D, controls the release of pro-inflammatory cytokines IL-1β and IL-18 [16,22,23,24], among others. Thus, due to its potential as a factor causing inflammation, it seems that GSDM D may be important in the diagnosis and treatment of patients with bladder cancer. Previous studies have indicated an increase in the expression of the GSDM D gene and protein in BC tissues. Moreover, higher expression of its gene and lymph node involvement have been associated with increased mortality [25]. However, it should be noted that, to the best of our knowledge, serum GSDM D has never been assessed in the serum of patients with BC. Therefore, the aim of this study was to evaluate both the significance and diagnostic utility of serum gasdermin D in BC. This study also included the assessment of C-reactive protein (CRP) as a marker of inflammation, as well as CA19-9 and CEA as complementary tumour markers. Although CEA and CA19-9 are associated with cancers other than BC, there are reports suggesting that their levels may change during the course of this malignancy. Thus, considering the fact that both CEA and CA19-9 are regarded as “universal” markers, we decided to assess and compare the diagnostic utility of CEA and CA19-9 with GSDM D.

## 2. Material and Methods

### 2.1. Study Design

This study involved patients with BC as well as a control group (C). Each patient in the study had their GSDM D concentrations measured. Additionally, the levels of CA19-9 and CEA, as comparative cancer biomarkers, were determined. The obtained results of the examined parameters were compared between the healthy group and BC patients, as well as between different stages of cancer.

### 2.2. Subjects

The study group consisted of 42 males and 8 females, with a mean age of 70.4 (min 42 and max 87). The patients diagnosed with BC were hospitalized in the Urology Department of the Medical University of Bialystok. BC was diagnosed based on cystoscopy, ultrasound, patient symptoms, and histopathological examination results. A total of 50 patients were scheduled for transurethral resection of bladder tumour (TURBT). Bladder tumours were identified through cystoscopy and ultrasound. No other active cancers were detected in these patients. Tumour staging and classification were carried out according to the guidelines of the European Association of Urology (EAU). BC patients were categorized into two subgroups based on the grade of tumour: high-grade (HG, 48%) and low-grade (LG, 52%). Moreover, the group of BC patients was divided according to TNM classification: Ta, T1, or T2.

### 2.3. Control Group

The mean age of healthy volunteers was 44.1 years (min 17 and max 79), and the group consisted of 17 females and 13 males. The following exclusion criteria were applied to potential participants in the control group: any comorbidities that could influence the concentration of tested parameters, pathological conditions of the urinary system, and active infections.

### 2.4. Ethical Procedures

All bladder cancer patients and healthy volunteers provided informed consent. On 25 June 2020 the study received approval from the Bioethical Committee at the Medical University of Bialystok (APK.002.240.220).

### 2.5. Samples

All blood samples were obtained through venipuncture. Serum specimens were collected through centrifugation, then aliquoted and stored at −80 °C until analysis.

### 2.6. Methods

The concentration of gasdermin D was measured using the ELISA method. The ELISA kit was provided by Biorbyt Ltd. (cat. no. orb1100903; Cambridge, UK). According to the manufacturer’s guidelines, duplicate measurements were performed for each standard, control, and sample.

According to the manufacturer’s instructions, all samples were diluted twice, so each result was multiplied by two.

The detection range of serum gasdermin D listed in the instructions equalled 0.156–10 ng/mL.

The levels of CA19-9, CEA, CRP, creatinine, and urea were determined using the Alinity analyzer (Abbott; Green Oaks, IL, USA) in accordance with the manufacturer’s guidelines. The details of the aforementioned measurements are presented in Table 1.

For concentrations below the detection limit, the lowest detectable values were applied.

All measurements were performed on the same day after thawing samples stored at −80 °C. All gradual thawed serum samples were thoroughly mixed on a vortex using low speed. After conducting control measurements at two levels, the concentrations of CEA, CA19-9, creatinine, and urea were determined using an automated method on Alinity analyzer. The GSDM D measurements were performed manually, and the results were read on an automated ETI-max 3000 reader (Diasornin, Saluggia, Italy). The room temperature where the measurements were performed was 21.5 °C, with humidity of approximately 50%. The lighting conditions for all measurements were standard, except during the substrate addition step when performing GSDM D measurements. According to the manufacturer’s recommendations, the substrate was added in a darkened room to minimize the impact of light on the measurement results.

### 2.7. Statistical Analysis

Statistica 13.3 was used to perform statistical analysis of results. The Mann–Whitney U test was used to evaluate differences between the groups, while the Spearman rank correlation test was applied to examine the relationship between variables. The optimal cut-off values for examined markers were calculated using the Youden index. Statistically significant differences were considered results with *p* < 0.05.

## 3. Results

### 3.1. Serum Concentrations of Gasdermin D and Tumour Markers

Serum concentrations of tested parameters in the serum of BC patients and the controls are shown in Table 2.

The serum concentrations of GSDM D showed a significant difference between the groups tested (*p* < 0.001). Likewise, the CRP, CEA, and CA19-9 levels were markedly elevated in BC patients compared to C (*p* = 0.004, *p* < 0.001, *p* = 0.047, respectively). In contrast, the urea and creatinine concentrations did not differ significantly between bladder cancer patients and controls (*p* = 0.077 and *p* = 0.275, respectively).

**Figure 1:**
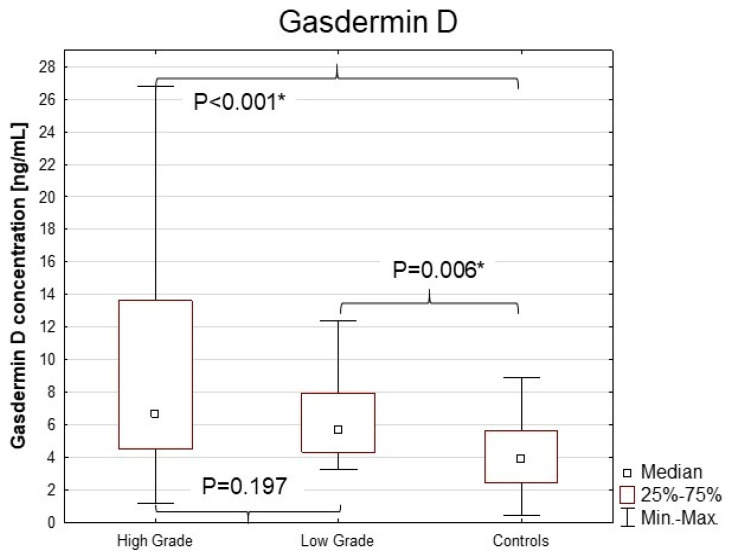
Serum concentrations of gasdermin D in high-grade and low-grade bladder cancer patients and the control group. Statistically significant differences between the groups are indicated by the * symbol.

The statistical analysis revealed that the serum concentrations of gasdermin D depend on the tumour grade (*p* < 0.001, H = 16.621) (Figure 1). The median of GSDM D concentration was significantly higher in low-grade (5.75) and high-grade (6.71) bladder cancer than in healthy controls (3.95; *p* = 0.006 and *p* < 0.001, respectively).

Moreover, the median concentrations of CEA were higher in low-(2.48) and high-grade (2.65) cancer in comparison to the control group (1.73; *p* = 0.001 for both). The median levels of GSDM and CEA did not differ between the low- and high-grade cancer (*p* = 1.000 for both). CRP and urea concentrations were elevated in high-grade (4.85 and 40.66, respectively) bladder cancer patients compared to the control group (1.25 and 29.96; *p* = 0.001 and *p* = 0.028, respectively). There were no differences in CRP and urea concentrations between HG and LG (1.40 and 32.10) (*p* = 0.197; *p* = 0.124, respectively) and controls (*p* = 1.000; *p* = 0.452, respectively). The CA19-9 and creatinine concentrations were similar among the control group (3.92 and 0.82), low-grade (6.55 and 0.82), and high-grade bladder cancer patients (5.42 and 0.89; *p* = 0.101, H = 4.586 and *p* = 0.133, H = 4.032, respectively).

The serum concentrations of CRP, CEA, CA19-9, urea, creatinine, and GSDM D in accordance with the tumour infiltration depth (T) are shown in Table 3.

The concentrations of GSDM D, CRP, CEA, CA19-9, urea, and creatinine were similar in the Ta, T1, and T2 groups (*p* = 1.000, H = 0.000; *p* = 0.060, H = 12.091; *p* = 1.000, H = 0.000; *p* = 1.000, H = 0.000; *p* = 0.533, H = 5.087; *p* = 0.110, H = 10.386, respectively).

The serum concentrations of CRP, CEA, CA19-9, and GSDM D according to the sex of the tested patients (a total of patients with bladder cancer and healthy individuals) were also evaluated (Table 4).

The median concentrations of CA19-9 were significantly higher in males than females (*p* = 0.035). The concentrations of GSDM D, CRP, and CEA were similar in both sexes (*p* = 0.116; *p* = 0.130; *p* = 0.280, respectively).

### 3.2. Correlations of Gasdermin D with Other Tested Parameters

Correlations between gasdermin D, CRP, CEA, CA19-9, creatinine, and urea are shown in Table 5.

Spearman’s rank correlation test demonstrated that GSDM D correlated with CRP and CEA concentrations. CRP also correlated with patients’ age and creatinine levels, while CEA correlated with patients’ age and CA19-9 concentration. Moreover, CA19-9 correlated with patients’ age and urea levels. Urea concentration correlated with patients’ age, but the strongest correlation was observed between urea and creatinine concentrations.

### 3.3. Diagnostic Power of GSDM D

Table 6 presents the diagnostic utility of tested biomarkers in bladder cancer. Gasdermin D showed a higher diagnostic sensitivity than CEA (83.8% vs. 81.3%) and slightly lower sensitivity compared to CA19-9 (83.8% vs. 89.6%), but the specificity for GSDM D and CA19-9 was moderate and identical (60.0%). Gasdermin D had the highest negative predictive value (75%) among all the tested markers. The highest positive predictive value and diagnostic accuracy were observed for CEA (84.8% and 79.8%, respectively), while the accuracy for gasdermin D was slightly lower (73.1%).

**Figure 2:**
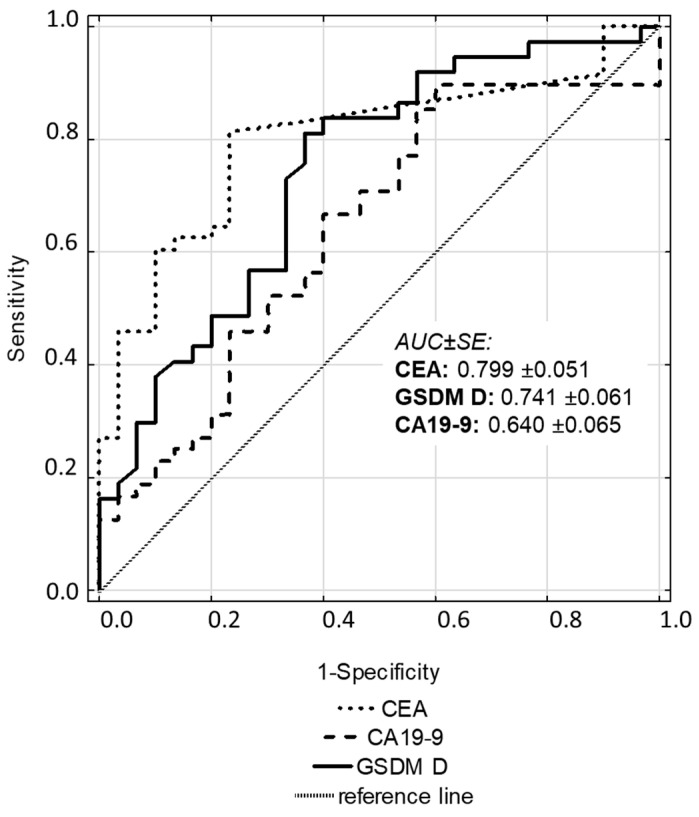
ROC curves for gasdermin D, CEA, and CA19-9 in bladder cancer. Captions: All the detailed conditions of the experiment are described in Section 2.

Gasdermin D has a moderate diagnostic accuracy with an AUC of 0.741, which suggests a reasonably good ability to differentiate between bladder cancer and non-cancerous conditions. The highest AUC was found for CEA, and the lowest AUC was found for CA19-9, indicating that CA19-9 is less effective in distinguishing between the tested conditions (Figure 2).

## 4. Discussion

Programmed cell death, also known as pyroptosis, is an actively induced form of cell death that involves cell disintegration and the release of molecules, including signalling proteins. Pyroptosis is most often described in the context of infectious disorders, because it attracts immune cells responsible for fighting pathogens and eliminates them [8,10]. The process of induced pyroptosis is controlled by gasdermins. Human cells produce six distinct gasdermins: A, B, C, D, E, and F. Each of the gasdermin molecules is made up of two components: the first one that triggers pyroptosis, and the second one that binds to the first to prevent it from causing cell death. Proteases are responsible for separating these two components and activating gasdermin, with caspase-1 being the most well-studied. Caspase-1 activates gasdermin D, among others, which is present in every cell of the body. It binds to the cell membrane, forming a pore that allows the release of molecules, thereby inducing pyroptosis [22,26,27,28].

Gasdermins in cancer is a topic that is generating increasing interest among scientists. It has been shown that in lung, breast, colorectal cancers, and hepatocellular carcinoma, gasdermins can affect the tumour microenvironment [29,30]. Uncontrolled activation of gasdermin can lead to chronic inflammation, which promotes the development of cancer. On the other hand, it has also been suggested that therapies aimed at modulating pyroptosis could aid in the treatment of both inflammation and cancer [30,31]. However, there is currently a lack of data on the role of GSDM D in the development and diagnosis of difficult-to-detect urinary tract cancers, including widespread bladder cancer.

Taking the above into account, the aim of our study was to assess the diagnostic significance of GSDM D in bladder cancer. To date, only a few studies have been published on GSDM D and bladder cancer. It has been observed that lower GSDM D gene expression in bladder cancer patients is associated with an increased risk of tumour recurrence and a higher mortality. Moreover, higher tissue expression in the muscle of invasive bladder cancer patients in comparison to the non-muscle invasive bladder cancer group has been noted; additionally, it was higher in comparison to the control group [25]. These results are in accordance with those noted by Peng et al., who found that elevated emmprin (CD147) expression promoted tumour proliferation in bladder cancer by activating GSDM D. This activation may subsequently serve as a negative prognostic marker. However, it should be noted that the direct and clear connection between GSDM D and CD147 is still unknown [32]. In addition, it has been shown that increased expression of GSDMB isoform-3 correlates with a favourable outcome in urothelial bladder cancer [33]. On the other hand, He et al. proposed that the increased expression of gasdermin B contributes to the promotion of bladder cancer growth. This can be explained by the fact that GSDM B binds to STAT3, thereby activating STAT3 signalling and modulating glucose metabolism and increasing its phosphorylation in bladder cancer. Contrary, the interaction between ubiquitin-specific peptidase 24 (USP24) and GSDMB prevents the degradation of gasdermin B in bladder cancer, and this may be a potential therapeutic target in the future [34].

However, according to our knowledge, this study is the first that evaluates the concentration of gasdermin D in the blood of patients with BC. We have demonstrated that the levels of gasdermin D are nearly double in the serum of BC patients compared to healthy controls. Additionally, the median concentration of GSDM D was significantly elevated in both low-grade and high-grade bladder cancer compared to healthy controls. However, the median levels of GSDM D were similar between the low- and high-grade cancer cases, and between different sizes and extents of tumour, classified in accordance to TNM classification.

It seems that gasdermin D play a fundamental role in the process of pyroptosis, which occurs at many stages of cancer development. As a result, the concentration of GSDM D may remain relatively constant at different stages of cancer advancement and regardless of the degree of malignancy. Additionally, we speculate that gasdermin D as a protein modulating the immune system response may contribute to the development of a chronic inflammatory state, which is one of the factors that may contribute to the development of cancer [35]. Therefore, if GSDM D is mainly involved in maintaining immune homeostasis, its concentration will not correlate with the stage of cancer advancement. The proof of this may be the fact that we observed positive correlation between GSDM D and CRP and CEA concentrations. Given that CEA levels can rise in chronic inflammation, and that CRP is an acute-phase protein [36,37], it can be concluded that GSDM D is closely associated with the inflammatory response during the progression of cancer. Going forward, we observed that CEA shows the best overall diagnostic performance with the highest AUC, followed by gasdermin D. GSDM D, as well as CEA, also offer good sensitivity, indicating their potential value in detecting bladder cancer.

The moderate diagnostic specificity of GSDM D, but high specificity of CEA, suggests that GSDM D could be more useful as a screening tool, but in combination with CEA to improve diagnostic accuracy.

## 5. Conclusions

This is the first study to assess the diagnostic relevance of serum gasdermin D in bladder cancer patients. We revealed that GSDM D concentrations are significantly elevated in bladder cancer, and it should be noted that its levels were higher in both low- and high-grade cancers in comparison to the control group. Furthermore, a relationship between GSDM D, CRP, and CEA could suggest the immune system’s involvement in cancer progression. In conclusion, GSDM D appears to be a valuable diagnostic biomarker, especially when used in conjunction with other biomarkers. Its high sensitivity, as well as its negative predictive value, make it useful for the early detection of bladder cancer.

## Figures and Tables

**Table 1:** Details of CA19-9, CEA, CRP, creatinine, and urea assays on the Alinity analyzer (Abbott).

Parameter	Method	Detection Range
CA19-9	chemiluminescent microparticle immunoassay (CMIA)	2.06–1200.00 U/mL
CEA	chemiluminescent microparticle immunoassay (CMIA)	1.73–1500.00 ng/mL
CRP	immunoturbidimetric	1.00–480.00 mg/dL
creatinine	enzymatic	2.5–400.00 mg/dL
urea	enzymatic	3.00–125.00 mg/dL

**Table 2:** The results of serum gasdermin D and CRP, CEA, CA19-9, urea, and creatinine concentrations.

	Variable Tested	GSDM D [ng/mL]	CRP [mg/dL]	CEA [ng/mL]	CA19-9 [U/mL]	Urea [mg/dL]	Creatinine [U/mL]
Bladder cancer (A)	Median (min–max values).	6.33 ^B^* (1.21–26.78)	2.05 ^B^* (1.00–103.00)	2.62 ^B^* (1.73–18.83)	6.37 ^B^* (2.05–256.43)	34.24 (17.12–79.18)	0.84 (0.51–2.13)
Control group (B)		3.95 ^A^* (0.49–8.87)	1.26 ^A^* (1.00–6.70)	1.73 ^A^* (0.59–4.26)	3.92 ^A^* (2.06–28.40)	29.96 (12.84–49.92)	0.82 (0.55–1.04)

^A^, Bladder cancer; ^B^, Control group; *, the significant differences between groups.

**Table 3:** Serum concentrations of tested parameters according to the tumour infiltration depth (T).

	Variable Tested	GSDM D [ng/mL]	CRP [mg/dL]	CEA [ng/mL]	CA19-9 [U/mL]	Urea [mg/dL]	Creatinine [U/mL]
Ta	Median (min–max values).	6.00 (3.54–12.37)	1.40 (1.00–103.00)	2.56 (1.73–12.16)	4.66 (2.05–74.06)	32.10 (17.12–47.08)	0.83 (0.51–1.14)
T1		6.50 (2.25–26.78)	4.85 (1.00–103.00)	4.03 (1.73–18.83)	9.65 (2.05–256.43)	34.24 (17.12–79.18)	1.00 (0.61–2.13)
T2		7.57 (4.74–18.79)	23.10 (3.00–68.40)	2.35 (1.73–3.00)	3.05 (2.13–29.83)	42.80 (23.54–51.36)	0.78 (0.68–0.91)

**Table 4:** Serum concentrations of GSDM D, CRP, CEA, and CA19-9 according to the sex of the patients.

	Variable Tested	GSDM D [ng/mL]	CRP [mg/dL]	CEA [ng/mL]	CA19-9 [U/mL]
Males	Median (min–max values)	5.49 (1.22–26.78)	1.50 (1.00–103.00)	2.13 (1.73–18.83)	6.66 (2.05–256.43)
Females		4.79 (0.49–18.79)	1.20 (1.00–103.00)	1.73 (0.59–12.00)	4.12 (2.05–19.08)

**Table 5:** Spearman correlations between tested variables in the total study group.

Total Study Group	GSDM D	CRP	CEA	CA19-9	Urea	Creatinine	Age
GSDM D							
r		0.278	0.341	0.159	−0.070	0.025	0.209
*p*		0.017 *	0.004 *	0.195	0.546	0.824	0.065
CRP							
r	0.278		0.110	0.157	0.115	0.276	0.244
*p*	0.017 *		0.237	0.093	0.155	<0.001 *	0.026 *
CEA							
r	0.341	0.110		0.252	0.002	−0.105	0.287
*p*	0.004 *	0.237		0.004 *	0.987	0.256	0.010 *
CA19-9							
r	0.159	0.157	0.252		0.224	0.142	0.288
*p*	0.195	0.093	0.004 *		0.018 *	0.126	0.010 *
Urea							
r	0.159	0.115	0.002	0.224		0.531	0.413
*p*	0.195	0.155	0.987	0.018 *		<0.001 *	<0.001 *
Creatinine							
r	0.025	0.276	−0.105	0.142	0.531		0.131
*p*	0.824	<0.001 *	0.256	0.126	<0.001 *		0.208
Age							
r	0.209	0.244	0.287	0.288	0.413	0.131	
*p*	0.065	0.026 *	0.010 *	0.010 *	<0.001 *	0.208	

Correlation ratio (r): 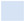, 0.000–0.300; 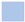, 0.301–0.400; 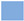, 0.401–0.500; 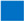, 0.501–0.700. *, significant correlation between tested variables.

**Table 6:** The diagnostic utility of GSDM D, CEA, and CA19-9 in bladder cancer.

	Cut-Off from the ROC	Sensitivity [%]	Specificity [%]	PPV [%]	NPV [%]	ACC [%]
GSDM D [ng/mL]	4.34	83.8	60.0	72.1	75.0	73.1
CEA [ng/mL]	1.74	81.3	76.7	84.8	71.9	79.8
CA19-9 [U/mL]	2.07	89.6	60.0	70.5	70.6	70.5

PPV, positive predictive value; NPV, negative predictive value; ACC, accuracy.

## Data Availability

The data that support the findings will be available on request under the corresponding author’s e-mail: monika.gudowska-sawczuk@umb.edu.pl.
